# Latitudinal trends in human primary activities: characterizing the winter day as a synchronizer

**DOI:** 10.1038/s41598-018-23546-5

**Published:** 2018-03-28

**Authors:** José María Martín-Olalla

**Affiliations:** Universidad de Sevilla, Departamento de Física de la Materia Condensada, PO Box 1065, ES41080 Seville, Spain

## Abstract

This work analyzes time use surveys from 19 countries (17 European and 2 American) in the middle latitude (38–61 degree) accounting for 45% of world population in this range. Time marks for primary activities are contrasted against light/dark conditions. The analysis reveals winter sunrise synchronizes labor start time below 54 degree, occurring within winter civil twilight. Winter sunset is a source of synchronization for labor end times. Winter terminator punctuate meal times in Europe: dinner occurs 3 h after winter sunset time within 1 h; 40% narrower than variability of dinner local times. The sleep-wake cycle of laborers is shown to be related to winter sunrise whereas standard population’s appears to be irrespective of latitude. The significance of the winter terminator depends on two competing factors average labor time (~7 h30 m) and the shortest photoperiod. Winter terminator gains significance when both roughly matches. That is within a latitude range from 38 degree to 54 degree. The significance of winter terminator as a source of synchronization is also related to contemporary year round time schedules: the shortest photoperiod represents the worst case scenario the society faces.

## Introduction

Earth’s rotation period (one day or *T* = 24 h, the definition of hour) notably influences biological rhythms^1,2^ and social human behavior. It is also the time basis to which mechanical clocks are sync. Clocks track time offset relative to the subsolar meridian— the great circle intersecting Earth’s rotation axis and the subsolar point, where the Sun is directly overhead— hence clock time is insensitive to latitude.

Earth’s orbit and the obliquity of the rotation axis (*ε* = 23.437°) seasonally alters the latitude of the subsolar point and makes the terminator —a great circle which separates light from darkness, with the subsolar point being one of its poles— characteristically differ from meridians, except when an equinox occurs or when yearly averaged values are considered. As a consequence clock readings are out of sync with the terminator: along a meridian day and night depend on latitude. The terminator shapes sunrises sunsets, and generally speaking the seasonal light/dark cycle, which is best noticed in the middle latitude range: between tropics and polar regions.

Human primary activity time marks (rise times, bed times, meal times, working times) are reported by the clock. It is an understanding that these time marks occur at prescribed values, within some variability linked to climate, cultural, legal, political or inherited habits. This understanding is a primary outcome of the worldwide use of time zones, which offset Earth’s rotation and accommodate the variability solar events and human activities measured by an universal clock. An acceptable value of the variability of time marks linked to primary activities is one hour, the standard subdivision of one day and the standard width of a physical time zone. In so doing we inadvertently understand human activity is sync to noon and the terminator would play no role in the description of these time marks because it does not play a role in clock-time either.

Daylight is the predominant synchronizer (zeitgeber) for the human clock^3^. Sleep activity can be traced by diurnal free preferences (circadian phenotypes or chronotype) which measure our preferred readiness by morningness-eveningness tests like the Horne-Östberg (HO) questionnaire^4^, the Composite Scale of Morningness^5^ (CSM) or the Munich ChronoType Questionnaire (MCTQ)^6^. For two of them (HO and CSM) the result is not given by a true metric of time, but a score. For MCTQ results are given through mid-sleep times, which are a metric of time. Either case their scores and questionnaires refer to clock time, instead of time distance to the terminator line, which, understandably, is a complicated metric to set up in a questionnaire. Understanding the role of latitude^7–10^ or latitude prone quantities like sunrise/sunset times, photoperiod or insolation, in morningness scores is then an open issue^11,12^ with interesting derivatives^13,14^.

Going beyond sleep-wake cycles the question to address in this work is to what extend are human primary activities sync to noon. Should people living along a meridian be doing these activities at the same time within some variability? An affirmative answer would result in meridional behavior of the activity. A negative answer would prompt a second question: would differences be systematic in latitude (the free parameter along a meridian)? Could they be linked to the non-meridional terminator?

These questions inspect whether ancient time reckoning synced to sunrises and sunsets, and sensitive to the seasonal cycle still plays a role in industrialized societies, highly tied to clocks synced to noon with only one source of seasonality: Daylight Saving Time (DST). It is only recently with clocks assisted by GPS systems (both usually embedded on mobile phones or tablets) that people can track sunrise, noon and sunset times on a device. However, as Sandford Fleming put forward at the session held on Oct. 14, 1884 in the International Meridian Conference^15^, people have been easily using clock time as a proxy for these events by learning what the clock ticks when they happen and making appropriate decisions —for instance when an alarm clock should be set for rising up— accordingly.

This work is aimed to an analysis of human primary activities time marks extracted from time use surveys in seventeen European countries and two American countries which cover 45% of the world population living in the middle latitude range from 38° to 61°. Yearly averaged daily rhythms of main activities will be analyzed to obtain time marks representative of the country population. These time marks will be systematically contrasted against the light/dark daily and seasonal cycles. Not limited to free preferences or the sleep-wake cycle, the work is focused to labor activity which should be specifically prone to the light/dark cycle. The winter day —that with the shortest photoperiod— will show up as a synchronizer, within some variability, of human activity in this range of latitudes. The shortest photoperiod —the worst case scenario year round— would force synchronization in contemporary societies with year round time schedules.

## Methods: Data Sets

Time use surveys^16^ are performed in many countries, chiefly OECD countries, with varying periodicity. Their aim is ascertaining when we do primary universal activities like sleeping, working or eating, and which fraction of a standard day an activity requires. Research on time budgets predates to late nineteenth century and has evolved so as to include comparisons among different classes of individuals^17^, the time evolution of societies^18^ or its response to economic turmoils^19^.

Two sets of data will be studied in this paper. On the first hand, microdata from national time use surveys, six in Europe and two in America, which are publicly available^20–22^ or which could be obtained from institutions through petitions^23–27^. European surveys were regionalized by NUTS^28^ level 1 scheme; American surveys were regionalized by provinces (Canada) and Census Divisions (United States). However, this work will not analyze data from non-contiguous regions in France (overseas departments), Spain (Canary Islands) and United States (Alaska and Hawaii). This paper will focus in primary activities (sleeping, working and eating) and the analysis will be enriched with the location “at home” and watching TV, an indoor leisure activity.

In this set only laborers in a week day will be analyzed because their daily preferences should be most socially coupled and, also, should be driven most by external conditions like the light/dark cycle. Notice that this study is complementary to circadian phenotypes, which track free preferences.

The number of respondents satisfying the condition lies in the range of five thousands within each participating country, except for Ireland (500), Denmark (2000) and United States, where a continuous, multiyear survey accounts for forty thousand respondents. It will be assumed that the data provided by the survey represents yearly average conditions as it integrates answers throughout the year, except for Irish and Danish surveys which did not expand along a calendar year. Respondents fill up a diary where a day is sliced into 144 time slots of ten minutes each, except Irish survey which make 96 time slots of one quarter of an hour each.

Highlighting differences between countries through time use surveys is a complicated issue because survey guidelines should be harmonized beforehand. That is the goal of the Harmonised European Time Use Survey^29^ (Hetus, here after) in Europe. The second set includes data retrieved from “main activities/time of day” pre-prepared tables available at Hetus^30^ webtool (https://www.h6.scb.se/tus/tus/Statistics.html) which accumulates results from surveys prior to 2005. Here the standard population subset (twenty to seventy four years old) is analyzed. For the purpose of comparison with national time use surveys the shares of employees’ in this subset ranges from 34% to 52% with median at 43%. Sample size amounts to tens of thousands of respondents in most of the cases, ranging from 38000 (Italy) to 5500 (Norway). Also it should be mentioned that Hetus provide no data on locations; hence the location “at home” could not be retrieved in this set.

In the following discussion geographical data of countries (latitude and longitude) will represent population weighted median values extracted from the database of cities with a population larger than 1000 inhabitants at http://www.geonames.org. The cast of countries geographically extends from 38° to 61° in median latitude (see Table 1). A Kolmogorov-Smirnov uniformity test does not reject (*p*-value = 0.59) that the distribution of latitudes is uniformly distributed from minimum to maximum value, meaning voids in the distribution are unlikely. In 2010 roughly 20% of world population lived in that range on either hemisphere and the cast of countries to be analyzed accounts for 45% of them. Data of population by latitude were obtained from the Global Rural-Urban Mapping Project, compiled by William Rankin at Yale University http://www.radicalcartography.net/index.html?histpop. Finally, the cast of countries is culturally narrow: all of them are Western countries with Christian heritage in the Northern Hemisphere. With the sole exceptions of Lithuania and Bulgaria they belong to OECD. All European countries, except Norway, belong to the European Union.

**Table 1:** Overview of geographical data for participating countries whose data were retrieved from national time use surveys (1) or Harmonised European Time Use Surveys (2).

Country	Label	Set	Latitude *ϕ*	Time offset *δ* = Δ −*λ*/Ω	Sunrise ${{\boldsymbol{t}}}_{{\boldsymbol{w}}}^{{\boldsymbol{\uparrow }}}$	Daytime *D*_*w*_	Sunset ${{\boldsymbol{t}}}_{{\boldsymbol{w}}}^{{\boldsymbol{\downarrow }}}$
*Europe ϕ* < 54°							
Spain	ESP	1, 2	40.4°	72 min	08:34	09 h17 m	17:50
Bulgaria	BGR	2	42.7°	21 min	07:50	09 h01 m	16:52
Italy	ITA	1, 2	43.6°	11 min	07:43	08 h55 m	16:39
Slovenia	SVN	2	46.1°	2 min	07:43	08 h37 m	16:21
France	FRA	1, 2	47.8°	50 min	08:39	08 h23 m	17:01
Belgium	BEL	2	50.9°	42 min	08:45	07 h55 m	16:40
Germany	DEU	2	51.0°	23 min	08:26	07 h53 m	16:20
Poland	POL	2	51.8°	−17 min	07:50	07 h46 m	15:36
United Kingdom	GBR	1, 2	52.3°	6 min	08:16	07 h40 m	15:56
Ireland	IRL	1	53.3°	25 min	08:41	07 h29 m	16:10
Average Variability	2s({*x*_*i*_}) 2s(*x*)		48.0° 9.1°	24 min 52 min	08:15 $51\,{\rm{\min }}$	08 h18 m 01 h16 m	16:32 01 h15 m
*Europe ϕ* > 54°							
Lithuania	LIT	2	54.9°	24 min	08:49	07 h10 m	15:59
Denmark	DNK	1	55.7°	13 min	08:43	07 h00 m	15:43
Latvia	LVA	2	56.9°	24 min	09:03	06 h42 m	15:45
Sweden	SWE	2	59.2°	−5 min	08:53	06 h05 m	14:58
Estonia	EST	2	59.4°	21 min	09:20	06 h02 m	15:22
Norway	NOR	2	59.9°	18 min	09:22	05 h52 m	15:14
Finland	FIN	2	61.1°	20 min	09:37	05 h27 m	15:04
Average Variability	2s({*x*_*i*_}) 2s(*x*)		58.2° 4.7°	16 min 20 min	09:07 $40\,{\rm{\min }}$	06 h20 m 01 h16 m	15:26 $46\,{\rm{\min }}$
*Europe all*							
Average Variability	2s({*x*_*i*_}) 2s(*x*)		52.2° 12.7°	21 min 41 min	08:36 01 h10 m	07 h29 m 02 h21 m	16:05 01 h32 m
*America*							
United States	USA	1	38.5°	8 min	07:24	09 h28 m	16:52
Canada	CAN	1	45.5°	17 min	07:56	08 h41 m	16:38
Average Variability	2s({*x*_*i*_}) 2s(*x*)		42.0° 9.9°	12 min 13 min	07:40 $46\,{\rm{\min }}$	09 h05 m 01 h06 m	16:45 $20\,{\rm{\min }}$
*All data*							
Average Variability	2s({*x*_*i*_}) 2s(*x*)		51.1° 13.8°	20 min 39 min	08:30 01 h15 m	07 h39 m 02 h26 m	16:09 01 h30 m

Latitude (rounded to one tenth of degree) and time offset (rounded to one minute) values are weighted population median values. Winter sunrise time ${t}_{w}^{\uparrow }$, winter daytime *D*_*w*_ and winter sunset time ${t}_{w}^{\downarrow }$ where computed through Equations (3), (4) and (5) using the listed values of latitude and time offset. Sunrise and sunset times are local times. Time zones are not listed. For each subset sample average value and the variability expressed as twice sample standard deviation are listed.

## Solar Elevation Angle and Time

Ambient light conditions are described by the the solar elevation angle *z* —the altitude of the Sun, the angle between the horizon and the center of the solar disc— which is given by^31^:

1 $$\sin (z)=\,\cos (\lambda -{\lambda }_{s})\,\cos \,\varphi \,\cos \,{\varphi }_{s}+\,\sin \,\varphi \,\sin \,{\varphi }_{s},$$

where *ϕ*, *λ* are the local latitude and longitude, and *ϕ*_*s*_, *λ*_*s*_ are subsolar latitude and longitude, where the Sun is overhead (*z* = 90°) and no shadow is cast. Notice that in Equation (1) the argument of the first cosine is the subsolar longitude offset relative to local longitude.

Subsolar longitude changes with Earth’s rotation at the rate of Earth’s angular rotation of speed Ω = 2*π*/*T* = 72.7 μ rads^−1^ = 15.0 ° h^−1^. The rotation speed scales longitude and time on Earth and allows to rewrite Equation (1) as:

2 $$\cos ({\rm{\Omega }}\tau )=\frac{\sin (z)-\,\sin \,\varphi \,\sin \,{\varphi }_{s}}{\cos \,\varphi \,\cos \,{\varphi }_{s}},$$

where *τ* is the time offset to local noon (*λ*_*s*_ = *λ*, *τ* = 0), usually known as mean solar time.

Since the inverse cosine function is even, and provided that the absolute value of the RHS is smaller than one, Equation (2) yields two solutions which are symmetrically disposed around *τ* = 0. One solution is the rising or morning solution; the other one is the setting or evening solution. The time elapsed between these solutions *D* is then:

3 $$D(z,\varphi ,{\varphi }_{s})=\frac{2}{{\rm{\Omega }}}\,{\cos }^{-1}\,(\frac{\sin \,z-\,\sin \,\varphi \,\sin \,{\varphi }_{s}}{\cos \,\varphi \,\cos \,{\varphi }_{s}}).$$

From this, setting and rising times can be expressed as:

4 $${\tau }_{\downarrow \uparrow }(z,\varphi ,{\varphi }_{s})=\frac{T\pm D(z,\varphi ,{\varphi }_{s})}{2},$$

where *τ* is now conveniently set to *T*/2 (that is 12) at noon.

For a critical elevation angle *z*_*c*_ = −0.83°, Equation (1) gives the terminator; Equation (3) gives daytime or photoperiod —the time elapsed from solar upper limb crossing the horizon upward to crossing it back downward—, and Equation (4) gives sunset and sunrise mean solar times. The critical *z*_*c*_ is non-zero due to Sun’s finite size and due to atmospheric refraction. The solution for *z*_*c*_ = 0° renders the terminator line as great circle and gently simplifies mathematics but will not be used here.

In the preceding equations the subsolar latitude changes yearly due to the obliquity of Earth’s rotation axis, leads to the seasons of the year, and carries the calendar date in these equations. Solstices occur when subsolar latitude is the furthest to observer latitude in winter *ϕ*_*s*_ = −sign(*ϕ*)*ε* or the closest to observer latitude in summer *ϕ*_*s*_ = sign(*ϕ*)*ε* and, in this simple model, drive extreme solutions for the equations: the earliest and the latest sunrises and sunsets of the year, and the longest or shortest photoperiod. All of these extreme solutions strongly depend on local latitude at middle latitudes. In contrast, yearly averaged values, as well as equinoctial (*ϕ*_*s*_ = 0) values, are nearly independent of the local latitude, except at polar latitudes.

Universal time (UT1) is conceptually a proxy for subsolar longitude to which is related by *T*/2 − *λ*_*s*_/Ω, where *λ*_*s*_ is given as an offset relative to UT1 prime meridian, the Greenwich meridian; UT1 is also mean solar time at this meridian. Local time *t* differs from UT1 by the time zone offset Δ which is usually given by a whole number of hours. Finally, mean solar time at any meridian differs from UT1 by *λ*/Ω, which is the signed elapsed time between *λ*_*s*_ = 0 and *λ*_*s*_ = *λ* following Earth’s rotation. As a result, mean solar time and local time are related by:

5 $$\tau =t-{\rm{\Delta }}+\frac{\lambda }{{\rm{\Omega }}}=t-\delta ,$$

where the time offset *δ* measures how much local noon is delayed (*δ* > 0) or advanced (*δ* < 0) from civil midday. Time zone offset Δ is usually chosen so that *δ* lies in the range −30 min to 30 min conforming a standard clock. However, in Belgium, France, Spain and the Canadian province of Saskatchewan (among others regions in the world) clocks are set one hour in advance so that *δ* ranges from 30 min to 90 min, a difference which must be taken into account when comparing time schedules and morningness-eveningness tests. Otherwise time schedules are reported abnormally delayed and morningness scores abnormally biased toward the eveningness.

Table 1 lists the relevant geographical data for the participating countries and the forthcoming analysis: weighted population median latitude and time offset. It also lists latest sunrise time ${t}_{w}^{\uparrow }$ and earliest sunrise time ${t}_{w}^{\downarrow }$ year round expressed as local time. Finally, it lists the values of the shortest photoperiod *D*_*w*_(*z*_*c*_, *ϕ*, -sign(*ϕ*)*ε*). Time values were computed using the equations in this section, fed with the listed values of latitude and time offset.

## Results

### Daily rhythms and relevant time marks

Relevant time marks for the budget of time can be retrieved from the so-called daily rhythms where the shares of the sample (or a specific subset of the sample) doing a prescribed activity are shown as a function of time on an average day. The daily rhythm provide an insight into activity rate performance. It integrates regionally, hourly, daily and yearly the myriad of decisions that shape the lives of individuals. It is an implicit understanding that these decisions are independent between national time use surveys and it is this country level average values that will be compared in the forthcoming analysis. Regional values, tied by social clock within a country, will not be compared.

**Figure 1:**
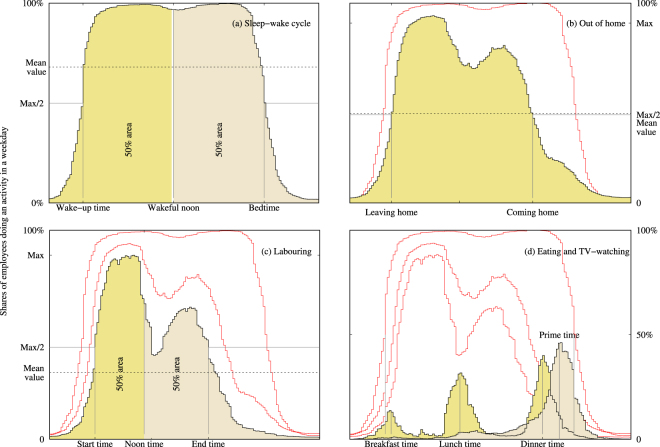
Daily rhythms and relevant time marks. Horizontal axes represent one day (24 h) starting at 4 am local time and vertical axes the shares of employees doing an activity at a given time on a weekday (Monday to Friday) in Italy as an example. From top to bottom and left to right: panel (a) shows the sleep-wake daily rhythm in filled style. Wakeful noon is the instant when half area has been consumed and half remains. Panel (b) mimics panel (a) but refers to the location “out of home”. The threshold defining leaving and coming home times are set to one half of the maximum value of the daily rhythm. The sleep-wake daily rhythm is displayed unfilled to provide context. Panel (c) shows in filled style the labor daily rhythm with the sleep-wake and out of home daily rhythms shown unfilled to provide context. Panel (d) shows eating and TV-watching daily rhythm in filled style; the remaining daily rhythms in unfilled style provide context. In panel (d) relevant time marks are determined from peak positions.

As an example Fig. 1 shows in fill style daily rhythms obtained from the Italian time use survey starting at 4 am and ending 24 later. The shaded area is the average daily consumption of the activity: the plot area amounts to one whole day in each panel. The mean value (panels a, b, and c) is the shares of employees that would have lead to the equivalent consumption if the activity were performed steadily, which is never the case. Mean value also measures daily consumption as a fraction of one day. The change of a daily rhythm with time shows net flux of people starting and stopping doing an activity at that time.

Start and end times can be computed on panels (a), (b) and (c) with the help of a threshold located at half the daily maximum rhythm. They characterize when a society is activated or deactivated from the point of view of an activity. On panel (d), eating and TV-watching activities occur in bursts. Peak positions will identify the relevant times. Eating has some specificities: first, surveys can not track energy intake so there is no difference in data between taking a snack or a proper meal. Secondly, in contrast with the daily rhythm shown on panel (d), the shares of population eating at a peak can be low enough so that the rhythm shows saw-tooth behavior, showing the preference for discrete, whole number of hours. For that reason eating daily rhythms coming from the Hetus webtool have all been smoothed and filtered with a Butterworth low-pass filter. Peaks were determined for the filtered signal. A visual inspection suffices to test that the algorithm retrieve well a time representative of each meal for every country.

A third time-mark will characterize sleep-wake and labor activity (panels a and c): activity noon time will be defined as the instant of time when half daily consumption has been burned and half remains. Wakeful noon is twelve hour apart from sleep noon, which slightly differ from mid-sleep.

The sleep-wake daily rhythm (panel a) resembles a rectangular function with two states (wakeful and asleep). Albeit for the statistical rounding effects at wake-up time and bedtime, sleep-wake daily rhythm is not different from the wakeful state of many individuals. This shows a confluence of individual decisions which is a primary observation of daylight (also a rectangular function) as a zeitgeber.

A fraction of awaken employees are “out of home” (panel b) at any hour of the day. Panel (b) shows this daily rhythm is sometimes far from a rectangular function: laborers can be at or out of home in a range of times and due to a myriad of reasons. One of these reasons is working (see panel c) whose daily rhythm is even more complex as it integrates morning, afternoon, night and split shifts in different ways.

In the morning, and quite generally, sleep-wake, out of home and labor daily rhythm soars very fast and with little time distance between them: employees get up, have breakfast, leave home and rush to get to work in a little time distance (see panel (c)). However, in the afternoon, the reverse process characteristically widens in time: each daily rhythm decreases at slower pace and their plunges are separated in time. That is an interesting difference with solar activity, which is perfectly symmetric around noon (see Equation (1)) on a daily basis.

### Time marks and latitude

A comprehensive analysis of human activity in relation to the returning phenomena of light and darkness is an open issue which must be related to longitude and latitude. The role of longitude is understood by converting local times into solar times through Equation (5) which takes into account Earth’s rotation and refers longitude to a common reference or meridian. Yet, the role of latitude is more complex.

**Figure 2:**
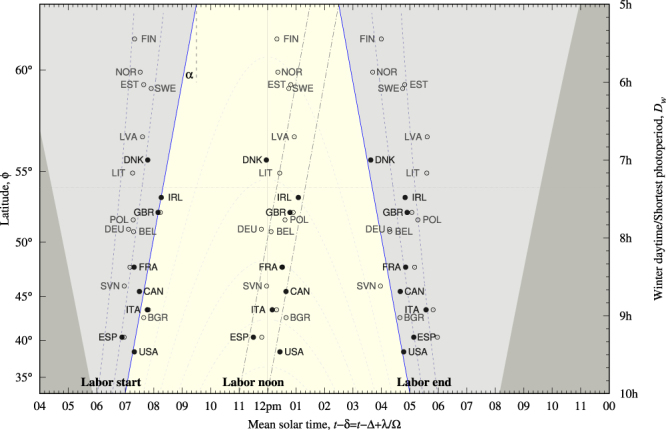
The daily and seasonal light/dark cycle and labor time marks versus winter daytime or latitude. Solid symbol stands for data extracted from national time use surveys; open symbols, for data computed from Hetus. Background colors display ambient light conditions. The lightest background displays the region where *z* > *z*_*c*_ (the solar limb is above local horizon) irrespective of calendar day. The darkest background displays the region where darkness prevails irrespective of calendar day (artificially delayed by one hour to accommodate daylight saving time). In the intermediate background color light and darkness depends on calendar date. The solid vertical line at 12 pm indicates solar noon and the horizontal dashed line is located at 54° latitude. Dashed lines shows a grid of constant *z* lines in the winter solstice. They are separated by 6° and start at −12° (nautical twilight), the outer most in the morning and evening. The slope *α* = −1/2 = −30 min/h for the winter sunrise time vs shortest photoperiod is noted. Dash-dotted straight lines shows 4 h to 5 h after winter sunrise. Labels indicate ISO-3166-1 alpha-3 country codes. Data are listed in Table 2. Bivariate correlations are reported in Table 3.

Figure 2 shows in graphic style the solutions to Equations (2) and (4) which shape ambient light conditions daily and year round. Notice that *x*-axis, mean solar time, runs in a Earth’s rotation period with noon happening at 12 pm (noted by a vertical line) irrespective of latitude. Vertical axes display latitude (left) and straight values of winter daytime (right) *D*_*w*_ = *D*(*z*_*c*_, *ϕ*, −sign(*ϕ*)*ε*) (see Equation (3)) which is the shortest photoperiod year round for a given latitudinal cline. In the forthcoming analysis *D*_*w*_ will play the role of a proxy for latitude. Notice that *D*_*w*_ decreases on increasing *ϕ* so that the right axis runs upside-down from the highest to the lowest values.

Background colors display ambient light conditions. The lightest background shows the region where light prevails irrespective of calendar date. It is bounded by the winter terminator, which is piece-wise linear in winter daytime (see Equation (4)) with slopes *α* = ±30 min/h (minutes of change in sunset or sunrise time per hour of change in photoperiod), a value to keep in mind. Seemingly, darkest background shows the region bounded by the summer terminator which sets the shortest night year round and has been delayed by one hour to simulate daylight saving time (a biannual, seasonal change in Δ), which is enforced in the regions analyzed in this paper with exceptions of Saskatchewan (Canada) and Arizona (United States). Intermediate background displays region where light or darkness seasonally alternates. Straight values of *D*_*w*_ make these regions have polygonal shape. Straight values of *ϕ* would have rendered them curvilinear as in any web page that shows a day/night map of the Earth.

Dashed lines display constant solar elevation angle at winter *z*_*w*_. Following Equation (2) noon and midnight are axes of symmetry for them. The outer most line is *z*_*w*_ = −12° —where nautical twilight starts or ends—followed by *z*_*w*_ = −6° —where winter civil twilight starts or ends. They characterize the significant change in ambient light conditions at dawn and dusk. The solid line in blue shows the terminator *z*_*w*_ = *z*_*c*_ = −0.83°. Afterwards lines are spaced by Δ*z*_*w*_ = 6° starting at *z*_*w*_ = 6°.

Upon this natural framework which shows up the daily and seasonal cycle of light and darkness Fig. 2 displays labor time marks, whose values are listed on Table 2. Notice that in the forthcoming analysis the independent variable *D*_*w*_ is placed on the *y*-axis, while the measured quantity *τ* is placed on the *x*-axis. Accordingly, slopes will be read relative to vertical, not horizontal, lines.

**Table 2:** Relevant parameters for labour start, noon and end points; *t* stands for local time; *τ* is mean solar time, Δ*t*_*w*_ is time distance to winter sunrise and *z*_*w*_ is the winter solar elevation angle at the event.

Country	Label	Labor Start			Labor Noon			Labor End		
		*t*	*τ*	*z*_*w*_	*t*	*τ*	Δ*t*_*w*_	*t*	*τ*	*z*_*w*_
*Time Use Surveys*										
United States	USA	07:25	07:20	0°	12:35	12:25	+05 h10 m	16:55	16:45	−1°
Spain	ESP	08:05	06:55	−6°	12:40	11:30	+04 h10 m	18:20	17:10	−6°
Italy	ITA	08:00	07:45	1°	12:20	12:10	+04 h40 m	17:45	17:35	−12°
Canada	CAN	07:45	07:30	−2°	12:55	12:40	+05 h00 m	16:55	16:40	−4°
France	FRA	08:10	07:20	−5°	13:20	12:30	+04 h40 m	17:40	16:50	−6°
United Kingdom	GBR	08:15	08:10	−1°	12:55	12:45	+04 h40 m	17:00	16:55	−9°
Ireland	IRL	08:45	08:15	−1°	13:30	13:00	+04 h45 m	17:15	16:45	−9°
Denmark	DNK	08:00	07:45	−5°	12:10	11:55	+03 h25 m	15:50	15:35	−2°
Average Variability	2s({*x*_*i*_}) 2s(*x*)	08:05 45 min	07:40 55 min	−2° 6°	12:50 55 min	12:25 01 h00 m	04 h35 m 0105 m	17:15 01 h30 m	16:50 01 h10 m	−6° 7°
*Hetus pre*-*prepared tables*										
Spain	ESP	08:10	07:00	−5°	13:00	11:50	+04 h25 m	19:10	18:00	−15°
Bulgaria	BGR	08:00	07:40	1°	13:00	12:40	+05 h10 m	17:00	16:40	−2°
Italy	ITA	08:00	07:50	2°	12:30	12:20	+04 h45 m	18:00	17:50	−14°
Slovenia	SVN	07:00	07:00	−7°	12:00	12:00	+04 h15 m	16:00	16:00	2°
France	FRA	08:00	07:10	−6°	13:20	12:30	+04 h40 m	18:00	17:10	−9°
Belgium	BEL	08:00	07:20	−7°	12:50	12:10	+04 h05 m	17:00	16:20	−3°
Germany	DEU	07:30	07:05	−8°	12:10	11:45	+03 h45 m	16:40	16:15	−3°
Poland	POL	07:00	07:15	−7°	12:20	12:35	+04 h30 m	17:00	17:15	−12°
United Kingdom	GBR	08:20	08:15	0°	13:00	12:55	+04 h45 m	17:10	17:05	−10°
Lithuania	LIT	07:40	07:15	−9°	12:50	12:25	+04 h00 m	18:00	17:35	−16°
Latvia	LVA	08:00	07:35	−7°	13:20	12:55	+04 h15 m	18:00	17:35	−16°
Sweden	SWE	07:50	07:55	−7°	12:40	12:45	+03 h45 m	16:40	16:45	−11°
Estonia	EST	08:00	07:40	−8°	13:10	12:50	+03 h50 m	17:10	16:50	−12°
Norway	NOR	07:50	07:30	−10°	12:40	12:20	+03 h20 m	16:00	15:40	−5°
Finland	FIN	07:40	07:20	−11°	12:40	12:20	+03 h05 m	16:20	16:00	−7°
Average Variability	2s({*x*_*i*_}) 2s(*x*)	07:50 45 min	07:25 45 min	−6° 8°	12:45 50 min	12:25 45 min	04 h10 m 01 h10 m	17:15 01 h45 m	16:50 01 h25 m	−9° 11°
*Both combined*										
Average Variability	2s({*x*_*i*_}) 2s(*x*)	07:55 45 min	07:30 50 min	−5° 8°	12:45 50 min	12:25 50 min	04 h20 m 01 h10 m	17:15 01 h40 m	16:50 01 h20 m	−8° 10°

Times have been rounded to the nearest five-minute mark except Irish data which have been rounded to the nearest quarter of an hour. Elevation angles have been rounded to the nearest whole degree. Countries are listed in increasing values of median latitude. Simple descriptive statistics values (sample average value and variability measured as twice sample standard deviation) are listed. Data are shown in Fig. 2. Correlations are reported on Table 3.

While data show some apparent trends with latitude, the most significant feature of labor start and end time marks is placement: in the neighborhood of the winter terminator despite they were obtained from yearly averaged labor daily rhythms. They do not stick to the equinoctial terminator (6 am/6 pm, irrespective of latitude) nor to the summer terminator: yearly averaged labor daily rhythms lean towards the winter terminator. Notice also that winter sunrise and summer sunset are just 12 h (solar time) apart (the same applies to summer sunrise and winter sunset); therefore it is placement, and the significance of winter and summer photoperiods, that allows to discriminate the role of the winter sunrise as a synchronizer of labor activity.

The full set of labor start times (sample size *N* = 23) shows variability 2*s*({*τ*_*i*_}) = 50 min —where *s* is sample standard deviation— smaller than one hour. However light conditions are different, the bulk of the sample (*N* = 16) lies below 54° latitude (see horizontal line in Fig. 2) where a bunch of labor start times is located around the winter civil twilight line and another pocket is located at the winter terminator. As a whole most of them are boxed within the winter civil twilight region *z* ∈ (−6°, 0°). Above 54° (*N* = 7) start labor times are boxed in the winter nautical region *z* ∈ (−12°, −6°).

End times run in a different fashion: variability rises to 2*s* = 1 h20 m and they can be boxed in a wider region of Δ*z* ~ 12° from winter sunset to the end of nautical twilight. Yet, in the case of end times the occurrence of solar angles (light conditions) are uniformly distributed above and below 54°. Table 2 lists *z*_*w*_ values for labor start and end times. The significance of these placements lies in the fact that they are describing the weakest values for *z* at labor start and end times year round.

Labor noon times are located close to solar noon where *z* changes with latitude and calendar date. The signature of the winter sunrise is revealed as data below 54° latitude can be boxed on one-hour strip centered 4 h30 m after winter sunrise, shown in Fig. 2.

Placement, boxing and the variability 2*s* are a key technique for understanding data but they do not exclude computing correlations. Bivariate correlations *τ* (a meridional quantity) vs *D*_*w*_ will punctuate trends with latitude, revealed by non-zero sample Pearson’s *r*^2^ coefficient and non-zero slope *p*. Also testing non-meridional quantities like *z*_*w*_ or Δ*t*_*w*_ (time distance to the winter terminator) vs *D*_*w*_ will help characterizing the trend since zero sample *r*^2^ and zero *p* will mean the tested property is stationary with latitude.

Table 3 reports bivariate correlations for labor time marks above and below *ϕ* = 54° and the full set of data. Weakest Pearsons’s *r*^2^ coefficients (lower correlations and greater stationarity) are italicized. Data below 54° show non-zero but low Pearson’s *r*^2^ = {0.231, 0.266, 0.0963} (start, noon, end) with slopes *p* = {−20.0(97), −20.7(92), 15(13)}min/h. In units of the winter terminator gradient |*α*| = 30 min/h, positive for the winter sunset, negative for winter sunrise, these slopes read *p*/*α* ~ {−2/3, −2/3, 1/2}. They are remarkable since the winter terminator is not the only source of the variance in labor times, understandably many other issues —cultural, economic, legal, geographical, our preferences for whole hours— will still play a role.

**Table 3:** Bivariate correlations for labor time marks and control variables.

*ϕ* < 54°	Labor Start	Labor Noon	Labor End
**Meridional quantity**		*y* = *τ*	
vs *x* = *D*_*w*_*r*^2^	0.231	0.266	0.0963
slope*p*(*u*_*p*_)	−20.0(97) min/h	−20.7(92) min/h	15(13) min/h
reference*y*_ref_	07:40	12:30	16:50
variability2*s*({*y*_*i*_})	55 min	55 min	01 h05 m
observations*N*	16	16	16
**Latitude prone quantity**	*y* = *z*_*w*_	*y* = Δ*t*_*w*_ (to sunrise)	*y* = *z*_*w*_
vs *x* = *D*_*w*_*r*^2^	*0.0461*	*0.067*	*0.0142*
slope*p*(*u*_*p*_)	1.1(14) °h^−1^	9.2(92) min/h	0.9(20) °h^−1^
reference*y*_ref_	−4°	+04 h30 m	−8°
variability2*s*({*y*_*i*_})	7°	$45\,{\rm{\min }}$	10°
observations*N*	16	16	16
*ϕ* > 54°			
**Meridional quantity**		*y* = *τ*	
vs *x* = *D*_*w*_*r*^2^	*8.43* × *10*^*−4*^	*0.0238*	0.192
slope*p*(*u*_*p*_)	0.6(99) min/h	−5(14) min/h	34(32) min/h
reference*y*_ref_	07:35	12:30	16:25
variability2*s*({*y*_*i*_})	$30\,{\rm{\min }}$	$40\,{\rm{\min }}$	01 h40 m
observations*N*	7	7	7
**Latitude prone quantity**			*y* = *z*_*w*_
vs *x* = *D*_*w*_*r*^2^			*0.0695*
slope*p*(*u*_*p*_)			−2.2(37)
reference*y*_ref_			−9°
variability2*s*({*y*_*i*_})			11°
observations*N*			7
*Full data set*			
**Meridional quantity**		*y* = *τ*	
vs *x* = *D*_*w*_*r*^2^	*0.0933*	0.138	0.165
slope*p*(*u*_*p*_)	−6.1(42) min/h	−7.7(42) min/h	13.3(66) min/h
reference*y*_ref_	07:30	12:25	16:55
variability2*s*({*y*_*i*_})	50 min	$50\,{\rm{\min }}$	01 h20 m
observations*N*	23	23	23
**Latitude prone quantity**			*y* = *z*_*w*_
vs *x* = *D*_*w*_*r*^2^			*0.0437*
slope*p*(*u*_*p*_)			0.89(91) °h^−1^
reference*y*_ref_			−8°
variability2*s*({*y*_*i*_})			10°
observations*N*			23

Top table refers observations below 54° latitude; middle, observations above that level; bottom, full data set. On each set the meridional quantity is labor mean solar times (*y* = *τ*) and the latitude prone quantity is winter solar elevation angle *z*_*w*_ or distance to winter sunrise Δ*t*_*w*_ at labor time marks. Either case the quantity is tested against shortest photoperiod *D*_*w*_. Each test reports Pearson’s *r*^2^ correlation coefficient (italicized in stationary results), slope and its uncertainty *p*(*u*_*p*_), a reference value *y*_ref_ at the level *D*_*w*_ = 8 h (*ϕ* ~ 50°) (top and bottom) or *D*_*w*_ = 6 h (*ϕ* ~ 60°) (middle) and the variability of the tested variable computed as twice its sample standard deviation. Uncertainties apply to the least two significant digits. Data are reported on Table 2 and shown in Fig. 2.

Testing *z*_*w*_ (at labor start and end times) and Δ*t*_*w*_ (at labor noon time) against *D*_*w*_ results in weaker Pearson’s *r*^2^ = {0.046, 0.067, 0.014}; uncertainties cover the zero-slope case. Labor noon time variability decreases 20% ($10\,{\rm{\min }}$) and variability of *z*_*w*_ is one twilight region (start) and one and a half (end).

All these evidences characterize the behavior of labor start and end time marks with latitude in the range 35° to 54°. The winter terminator arises as a synchronizer for labor start and end times in this range. Start times seem to be stronger synchronized to winter sunrise than end times do to winter sunset. Reasonably at labor start times societies have come from a common reference state and cultural differences are less significant than nine hours later at labor end times. On the other hand it should be mentioned a major problem this analysis faces: the span of the independent variable *D*_*w*_ in the range 35° to 54° (~2 h see Table 1) times *α* is roughly 1 h, the default variability in the dependent variable *τ*.

Above 54° the scenario differs: twilight zone opens and start becoming indistinguishable from meridians, also *D*_*w*_ falls well below the average labor time and sample size is small (*N* = 7). Table 3 reports bivariate correlations for labor times above 54°. Start times and noon times behave meridionally (*r*^2^ = {8 × 10^−4^, 0.0238} for *τ* vs *D*_*w*_ bivariate correlations) while end times find non-meridional behaviour as *z*_*w*_ vs *D*_*w*_ is stationary *r*^2^ = 0.0695. The full set of data (*N* = 23) mimics this pattern: start and noon times showing meridional behaviour and end times showing non-meridional behaviour. These observations suggest that above 54°, with photoperiod increasingly shorter than 7 h, laborers would find accommodation by advancing start times relative to sunrise so that they stack vertically in Fig. 2 with those of lower latitude, helping decrease the variability of labor start times. A daily outcome of this preference would be the advance of end times so that they get closer to the winter sunset and twilight lines throughout the full range of observed latitudes increasing the variability of labor end times.

The preceding analysis can be mimicked for the remaining primary activities with some complexities. First bold symbols refer to employees’ statistics while light symbols refer to standard population statistics coming from Hetus webtool. The standard population has a varying shares of non-employees within each country ranging from 48% to 66% of sample size. It is also an issue that Hetus webtool does not provide the sleep-wake daily rhythm. Instead it provides the “sleep and other personal care” daily rhythm. On this circumstance one should expect that start time marks would be slightly delayed: if an individual wake up and start taking personal care —for instance, washing up— she would not have changed her status in the daily rhythm until she finished doing personal care. For the same reason end times may be slightly advanced if individuals perform some personal care just before bedtime.

**Figure 3:**
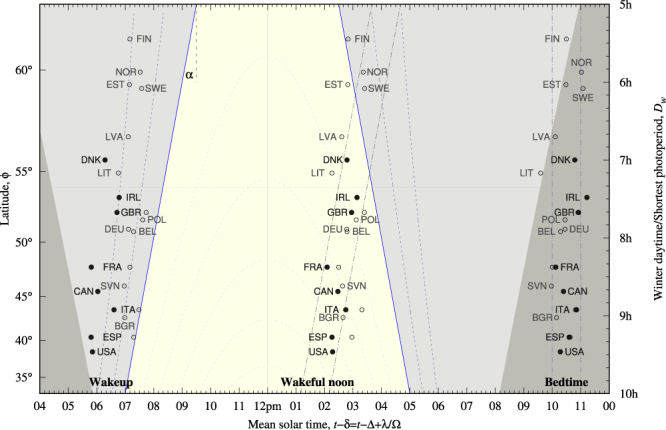
The same background as in Fig. 2 and the sleep-wake solar time marks versus latitude. From left to right: wake-up time, wakeful noon and bedtime. Solid symbols show data extracted from time use surveys for employees only; open symbols data from Hetus for the standard population. The slanted dash-dotted lines display a strip one hour centered 6 h35 m after winter sunrise. Data are listed in Table 4. Bivariate correlations are reported in Table 5.

Table 4 lists time marks for the sleep-wake cycle which are presented in Fig. 3. Morning times are dominated by the winter sunrise trend if data are grouped in three categories: standard population below 54° (*N* = 9), standard population above 54° (*N* = 6) and employees (*N* = 8, all but Denmark below 54°). Wakeful noon times of employees also look related to the winter sunrise trend as their data can be boxed in one-hour strip centered at 6 h35 m, shown in Fig. 3. Bedtimes can be boxed in a one-hour meridional (vertical) strip suggesting little dependence with latitude. Nonetheless, standard population data above 54° may exhibit a trend.

**Table 4:** Relevant parameters for wakeup time, wakeful noon time and bedtime; *t* stands for local time; *τ* is mean solar time, Δ*t*_*w*_ is time distance to winter sunrise and *z*_*w*_ is the winter solar elevation angle at the event.

Country	Label	Wake up			Wakeful noon			Bedtime	
		*t*	*τ*	*z*_*w*_	*t*	*τ*	Δ*t*_*w*_	*t*	*τ*
*Time Use Surveys* (*employees in weekday only*)									
United States	USA	06:00	05:50	−16°	14:25	14:15	+07 h00 m	22:25	22:15
Spain	ESP	07:00	05:50	−17°	15:30	14:15	+06 h55 m	23:50	22:35
Italy	ITA	06:45	06:35	−10°	14:55	14:45	+07 h15 m	23:00	22:50
Canada	CAN	06:20	06:00	−16°	14:45	14:30	+06 h50 m	22:40	22:25
France	FRA	06:40	05:50	−19°	14:55	14:05	+06 h15 m	22:55	22:05
United Kingdom	GBR	06:50	06:45	−12°	15:05	14:55	+06 h45 m	23:00	22:55
Ireland	IRL	07:15	06:45	−12°	15:30	15:15	+07 h00 m	23:45	23:15
Denmark	DNK	06:30	06:15	−17°	15:00	14:35	+06 h45 m	23:05	22:40
Average Variability	2s({*x*_*i*_}) 2s(*x*)	06:40 45 min	06:15 50 min	−15° 6°	15:00 45 min	14:35 40 min	06 h45 m 40 min	23:05 55 min	22:40 45 min
*Hetus pre*-*prepared tables* (*standard population*)									
Spain	ESP	08:30	07:20	−1°	16:10	15:00	+07 h35 m	23:50	22:40
Bulgaria	BGR	07:20	07:00	−6°	15:00	14:40	+07 h10 m	22:30	22:10
Italy	ITA	07:40	07:30	−1°	15:30	15:20	+07 h45 m	23:00	22:50
Slovenia	SVN	07:00	07:00	−7°	14:40	14:40	+06 h55 m	22:00	22:00
France	FRA	08:00	07:10	−6°	15:20	14:30	+06 h40 m	22:50	22:00
Belgium	BEL	08:00	07:20	−7°	15:30	14:50	+06 h45 m	23:00	22:20
Germany	DEU	07:30	07:05	−8°	15:10	14:45	+06 h45 m	22:50	22:25
Poland	POL	07:20	07:35	−5°	14:50	15:05	+07 h00 m	22:10	22:25
United Kingdom	GBR	07:50	07:45	−4°	15:30	15:25	+07 h15 m	23:00	22:55
Lithuania	LIT	07:10	06:45	−13°	14:40	14:15	+05 h50 m	22:00	21:35
Latvia	LVA	07:30	07:05	−11°	15:00	14:35	+05 h55 m	22:30	22:05
Sweden	SWE	07:30	07:35	−9°	15:20	15:25	+06 h25 m	23:00	23:05
Estonia	EST	07:30	07:10	−12°	15:10	14:50	+05 h50 m	22:50	22:30
Norway	NOR	07:50	07:30	−10°	15:40	15:20	+06 h20 m	23:20	23:00
Finland	FIN	07:30	07:10	−13°	15:10	14:50	+05 h35 m	22:50	22:30
Average Variability	2s({*x*_*i*_}) 2s(*x*)	07:3545 min	07:1535 min	−7°7°	15:1550 min	14:5545 min	06 h40 m01 h20 m	22:4501 h00 m	22:2550 min
*Both combined*									
Average Variability	2s({*x*_*i*_}) 2s(*x*)							22:55 01 h00 m	22:30 50 min

Note wakeful noon time is twelve hours apart from sleep noon time. Hetus data were obtained from the “sleep and other personal care” daily rhythm. Times have been rounded to the nearest five-minute mark except Irish data which have been rounded to the nearest quarter of an hour. Elevation angles have been rounded to the nearest whole degree. Simple descriptive statistic values (sample average value and twice sample standard deviation) are listed. Data are shown in Fig. 3. Bivariate correlations are reported on Table 5.

Results on bivariate correlations for the sleep/wake cycle are listed in Table 5. Employees’ wake up time marks report *r*^2^ = 0.36 and *p* = −16.9(92) min/h, roughly 3/5 of *α*. Correlations *z*_*w*_ vs *D*_*w*_ show smaller correlation *r*^2^ = 0.0175 with *z*_*w*_ irrespective of latitude and close to −15° and variability equal to one twilight region (6°). Therefore wake up conditions for employees are largely related to labor start times, synchronized by the winter sunrise. Wakeful noon times and bedtimes report similar values of *r*^2^, *p* and 2*s* when *τ* and Δ*t*_*w*_ are tested. Hence it is difficult to elucidate which synchronizer is stronger.

**Table 5:** Bivariate correlations for sleep-wake time marks and control variables and subsets.

*Employees*	Wake up	Wakeful noon	Bedtime
**Meridional quantity**		*y* = *τ*	
vs x = *D*_*w*_r^2^	0.36	0.478	0.329
slope*p(u*_*p*_*)*	−16.9(92) min/h	−17.2(74) min/h	−14.1(82) min/h
reference*y*_ref_	06:20	14:40	22:45
variability2*s*({*y*_*i*_})	$50\,\,{\rm{\min }}$	$45\,\,{\rm{\min }}$	$45\,\,{\rm{\min }}$
observationsN	8	8	8
**Latitude prone quantity**	*y* = *z*_*w*_	*y* = Δ*t*_*w*_ (to sunrise)	*y* = Δ*t*_*w*_ (to sunrise)
vs *x* = *D*_*w*_*r*^2^	*0.0175*	0.334	0.383
slope*p*(*u*_*p*_)	−0.5(14) °h^−1^	12.7(74) min/h	$\mathrm{15.8(82)}\,{\rm{\min }}/{\rm{h}}$
reference*y*_ref_	−15°	+06 h40 m	−09 h15 m
variability2*s*({*y*_*i*_})	6°	$40\,\,{\rm{\min }}$	$45\,\,{\rm{\min }}$
observations*N*	8	8	8
*Hetus ϕ* < 54°			
**Meridional quantity**		*y* = *τ*	
vs *x* = *D*_*w*_*r*^2^	0.207	*0.0441*	*0.0137*
slope*p*(*u*_*p*_)	$-\mathrm{12.2(91)}\,{\rm{\min }}/{\rm{h}}$	$-\mathrm{7(12)}\,{\rm{\min }}/{\rm{h}}$	$-\mathrm{4(13)}\,{\rm{\min }}/{\rm{h}}$
reference*y*_ref_	07:20	14:55	22:25
variability2*s*({*y*_*i*_})	$30\,\,{\rm{\min }}$	$40\,\,{\rm{\min }}$	$40\,\,{\rm{\min }}$
observations*N*	9	9	9
**Latitude prone quantity**	*y* = *z*_*w*_		
vs *x* = *D*_*w*_*r*^2^	0.224		
slope*p*(*u*_*p*_)	1.9(14) °h^−1^		
reference*y*_ref_	−6°		
variability2*s*({*y*_*i*_})	5°		
observations*N*	9		
*Hetus ϕ* > 54°			
**Meridional quantity**		*y* = *τ*	
vs *x* = *D*_*w*_*r*^2^	0.401	0.443	0.566
slope*p*(*u*_*p*_)	$-\mathrm{18(11)}\,{\rm{\min }}/{\rm{h}}$	$-\mathrm{28(16)}\,{\rm{\min }}/{\rm{h}}$	$-\mathrm{41(18)}\,{\rm{\min }}/{\rm{h}}$
reference*y*_ref_	07:15	15:00	22:35
variability2*s*({*y*_*i*_})	$35\,\,{\rm{\min }}$	$55\,\,{\rm{\min }}$	01 h05 m
observations*N*	6	6	6
**Latitude prone quantity**	*y* = *z*_*w*_	*y* = Δ*t*_*w*_ (to sunrise)	*y* = Δ*t*_*w*_ (to sunrise)
vs *x* = *D*_*w*_*r*^2^	*0.0607*	*0.00186*	*0.0872*
slope*p*(*u*_*p*_)	−0.6(13) °h^−1^	$\mathrm{1(16)}\,{\rm{\min }}/{\rm{h}}$	$-\mathrm{11(18)}\,{\rm{\min }}/{\rm{h}}$
reference*y*_ref_	−11°	+06 h00 m	−10 h25 m
variability2*s*({*y*_*i*_})	3°	$40\,\,{\rm{\min }}$	$45\,\,{\rm{\min }}$
observations*N*	6	6	6

Top table reports data for employees; next standard population (Hetus) below and above 54°. The meridional quantity is mean solar time at time marks (*y* = *τ*) and the latitude prone quantity is winter solar elevation angle *z*_*w*_ or distance to winter sunrise Δ*t*_*w*_ at sleep-wake time marks. Either case the quantity is tested against shortest photoperiod *D*_*w*_. Each test reports Pearson’s *r*^2^ correlation coefficient (italicized in stationary results), slope and its uncertainty *p*(*u*_*p*_), a reference value *y*_ref_ at the level *D*_*w*_ = 8 h (*ϕ* ~ 50°) and the variability of the tested variable computed as twice its sample standard deviation. Uncertainties apply to the least two significant digits.

Bivariate correlations for standard population tell a different history. Below 54° it is difficult to assess which synchronizer dominates wake up times. On the contrary, wakeful noon times and bedtimes are dominated by the meridional synchronizer as Pearson coefficients report low values *r*^2^ = {0.0441, 0.0137} with slopes *p* = {−7(12), −4(13)}min h^−1^ including *p* = 0 in the error band. Above 54° the three sleep/wake time marks appear to be synchronized by the winter sunrise: notice for instance the low variability in the latitude prone quantity.

Notwithstanding this, visual inspections of bedtimes in Table 4 or Fig. 3 suggest that they are similar for both employees and standard population. The full set of data results in variability 2*s*({*τ*_*i*_}) = 50 min so that they can be boxed in a one-hour strip located at 10 pm–11 pm (shown in Fig. 3), advocating that they might be globally irrespective of latitude at this range. Bivariate correlations *τ* vs *D*_*w*_ for data below 54° result in *r*^2^ = 0.0983 and *p* = −10.5(85) min/h (results not shown in Table 5). In any case a hypotheses concerning employees’ bedtimes faces the problem of low sample size in this analysis.

The argument on bedtimes is enlightened by inspecting TV prime time marks, the main activity preceding bedtime. Table 6 lists data for TV prime time marks which are similar for laborers and standard population and show little variability 2*s*({*τ*_*i*_}), smaller than one hour. Table 7 reports data on bivariate correlations *D*_*w*_ vs *τ* for employees and standard population below and above 54°. TV prime time marks above 54° display correlations when *τ* is tested against *D*_*w*_ but they are reduced if Δ*t*_*w*_ is tested against *D*_*w*_; variability 2*s* also shrinks 20% to 45 min. This suggests that TV prime time marks, like bedtimes, exhibit the winter sunrise signature above 54°. Below 54° TV prime time marks show no pattern with latitude as deduced from low Pearson coefficient, slope and variability in Table 7.

**Table 6:** Prime time mark obtained from the peak position of the daily watching TV daily rhythm.

Country	Label	TV Prime Time	
		*t*	*τ*
*Time Use Surveys* (*employees in weekday only*)			
United States	USA	20:45	20:40
Spain	ESP	22:45	21:35
Italy	ITA	21:45	21:35
Canada	CAN	21:30	21:15
France	FRA	21:45	20:55
United Kingdom	GBR	21:30	21:25
Ireland	IRL	22:30	22:00
Denmark	DNK	21:30	21:15
Average Variability	2s({*x*_*i*_}) 2s(*x*)	21:45 01 h15 m	21:20 50
*Hetus pre*-*prepared tables* (*standard population*)			
Spain	ESP	22:40	21:30
Bulgaria	BGR	21:30	21:10
Italy	ITA	21:40	21:30
Slovenia	SVN	20:50	20:50
France	FRA	21:50	21:00
Belgium	BEL	21:50	21:10
Germany	DEU	21:20	20:55
Poland	POL	20:50	21:05
United Kingdom	GBR	21:40	21:35
Lithuania	LIT	20:40	20:15
Latvia	LVA	20:50	20:25
Sweden	SWE	21:20	21:25
Estonia	EST	21:10	20:50
Norway	NOR	21:20	21:00
Finland	FIN	21:40	21:20
Average Variability	2s({*x*_*i*_}) 2s(*x*)	21:25 01 h00 m	21:05 45
*Both combined*			
Average Variability	2s({*x*_*i*_}) 2s(*x*)	21:30 01 h10 m	21:10 50

The table lists local time (*t*) and mean solar time (*τ*). Simple descriptive statistic values (sample average value and twice sample standard deviation) are listed. Times have been rounded to the nearest five-minute mark except Irish data which have been rounded to the next quarter of an hour. Bivariate correlations are reported on Table 6.

**Table 7:** Bivariate correlations for TV prime time marks and control variables for: top, employees (excluding USA); middle, standard population below 54°; bottom, standard population above 54°.

Employees	Prime Time
**Meridional quantity**	*y* = *τ*
vs *x* = *D*_*w*_*r*^2^	*0.0165*
slope*p*(*u*_*p*_)	$-\mathrm{3(12)}\,\,{\rm{\min }}/{\rm{h}}$
reference*y*_ref_	21:25
variability2*s*({*y*_*i*_})	$40\,\,{\rm{\min }}$
observations*N*	7
*Hetus ϕ* < 54°	
**Meridional quantity**	*y* = *τ*
vs *x* = *D*_*w*_*r*^2^	*0.0558*
slope*p*(*u*_*p*_)	$-\mathrm{7.8(87)}\,\,{\rm{\min }}/{\rm{h}}$
reference*y*_ref_	21:20
variability2*s*({*y*_*i*_})	$45\,\,{\rm{\min }}$
observations*N*	16
*Hetus ϕ* > 54°	
**Meridional quantity**	*y* = *τ*
vs *x* = *D*_*w*_*r*^2^	0.324
slope*p*(*u*_*p*_)	$-\mathrm{24(16)}\,\,{\rm{\min }}/{\rm{h}}$
reference*y*_ref_	21:05
variability2*s*({*y*_*i*_})	$55\,\,{\rm{\min }}$
observations*N*	7
**Latitude prone quantity**	*y* = Δ*t*_*w*_ (to sunrise)
vs *x* = *D*_*w*_*r*^2^	*0.0256*
slope*p*(*u*_*p*_)	$\mathrm{6(16)}\,{\rm{\min }}/{\rm{h}}$
reference*y*_ref_	+12 h05 m
variability2*s*({*y*_*i*_})	$45\,\,{\rm{\min }}$
observations*N*	7

The meridional quantity is mean solar times (*y* = *τ*) the latitude prone quantity is distance to winter sunrise Δ*t*_*w*_ at TV prime time mark. Either case the quantity is tested against shortest photoperiod *D*_*w*_. Each test reports Pearson’s *r*^2^ correlation coefficient (italicized in stationary results), slope and its uncertainty *p*(*u*_*p*_), a reference value *y*_ref_ at the level *D*_*w*_ = 8 h (*ϕ* ~ 50°) or *D*_*w*_ = 6 h (bottom) and the variability of the tested variable measured as twice its sample standard deviation. Last two items were rounded to the nearest five-minute. Uncertainties apply to the least two significant digits. Data are listed in Table 6.

**Figure 4:**
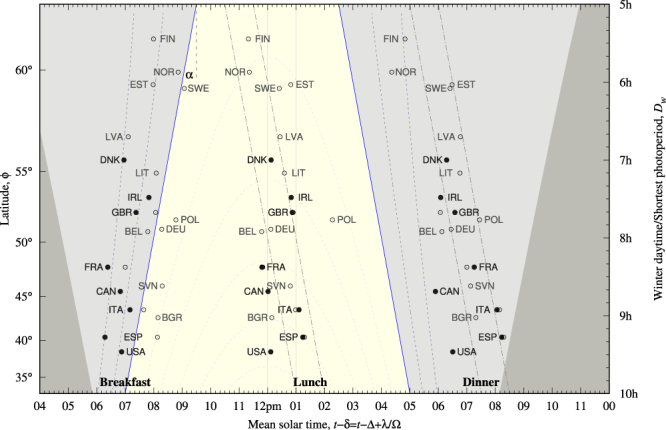
The same as in Figs 2 or 3 but with eating time marks: breakfast (left), lunch (center) and dinner (right). Solid symbols show data extracted from time use surveys and refer to laborers in a week day. Open symbols (Hetus) refer to the standard set of population. Dash-dotted lines display two bands of one hour width. The earliest band is centered at three and half hours before winter sunset. The latest band is centered at three hours after winter sunset. They enlighten the comparison of Δ*t*_min_ in Table 8. Bivariate correlations are reported on Table 9.

Figure 4 shows the relevant times associated with the eating daily rhythm, data are listed in Table 8. Breakfast times are largely different for solid symbols (employees) and open symbols (standard population) in the few cases for which both statistics have been analyzed. In contrast, lunch and dinner time marks apparently do not differentiate one from the other. Both of them exhibits an interesting pattern with latitude. European dinner times can be boxed in a one-hour strip located 3 h after winter sunset highlighting the role of the winter sunset as a synchronizer of dinner times. On the other hand lunch times can be equally boxed meridionally (vertically) in the hour following solar noon or in a one-hour strip centered 03 h30 m before winter sunset, suggesting competing synchronizers.

**Table 8:** Relevant parameters for breakfast, lunch and dinner.

Country	Label	Breakfast			Lunch			Dinner		
		*t*	*τ*	*z*_*w*_	*t*	*τ*	Δ*t*_*w*_	*t*	*τ*	Δ*t*_*w*_
*Time Use Surveys* (*employees in weekday only*)										
United States	USA	07:00	06:55	−5°	12:15	12:05	−04 h35 m	18:40	18:30	+01 h45 m
Spain	ESP	07:30	06:15	−12°	14:25	13:15	−03 h25 m	21:25	20:15	+03 h35 m
Italy	ITA	07:20	07:10	−4°	13:15	13:05	−03 h20 m	20:15	20:05	+03 h35 m
Canada	CAN	07:05	06:50	−8°	12:20	12:00	−04 h20 m	18:10	17:55	+01 h35 m
France	FRA	07:15	06:25	−13°	12:40	11:50	−04 h25 m	20:05	19:15	+03 h05 m
United Kingdom	GBR	07:30	07:25	−7°	13:00	12:50	−03 h00 m	18:40	18:35	+02 h45 m
Ireland	IRL	08:15	07:45	−4°	13:15	12:45	−03 h00 m	18:30	18:00	+02 h15 m
Denmark	DNK	07:10	06:55	−12°	12:20	12:05	−03 h25 m	18:30	18:15	+02 h45 m
Average Variability	2s({*x*_*i*_}) 2s(*x*)	07:25 45 min	07:00 01 h00 m	−8° 7°	12:55 01 h30 m	12:30 01 h05 m	03 h40 m 01 h20 m	19:15 02 h20 m	18:50 01 h45 m	02 h40 m 01 h30 m
*Hetus pre*-*prepared tables* (*standard population*)										
Spain	ESP	09:20	08:10	6°	14:30	13:20	−03 h20 m	21:30	20:20	+03 h40 m
Bulgaria	BGR	08:30	08:10	5°	12:30	12:10	−04 h20 m	19:40	19:20	+02 h50 m
Italy	ITA	07:50	07:40	0°	13:10	13:00	−03 h30 m	20:20	20:10	+03 h40 m
Slovenia	SVN	08:20	08:20	4°	12:50	12:50	−03 h30 m	19:10	19:10	+02 h50 m
France	FRA	07:50	07:00	−8°	12:40	11:50	−04 h20 m	19:50	19:00	+02 h50 m
Belgium	BEL	08:30	07:50	−3°	12:30	11:50	−04 h10 m	18:50	18:10	+02 h10 m
Germany	DEU	08:40	08:15	1°	12:30	12:05	−03 h50 m	18:50	18:25	+02 h30 m
Poland	POL	08:30	08:45	4°	14:00	14:15	−01 h35 m	19:10	19:25	+03 h35 m
United Kingdom	GBR	08:10	08:05	−2°	13:00	12:55	−02 h55 m	18:10	18:05	+02 h15 m
Lithuania	LIT	08:30	08:05	−3°	13:00	12:35	−03 h00 m	19:10	18:45	+03 h10 m
Latvia	LVA	07:30	07:05	−11°	12:50	12:25	−02 h55 m	19:10	18:45	+03 h25 m
Sweden	SWE	09:00	09:05	0°	12:20	12:25	−02 h40 m	18:20	18:25	+03 h20 m
Estonia	EST	08:20	08:00	−6°	13:10	12:50	−02 h10 m	18:50	18:30	+03 h30 m
Norway	NOR	09:10	08:50	−2°	11:40	11:20	−03 h35 m	16:40	16:20	+01 h25 m
Finland	FIN	08:20	08:00	−7°	11:40	11:20	−03 h25 m	17:10	16:50	+02 h05 m
Average Variability	2s({*x*_*i*_}) 2s(*x*)	08:25 01 h00 m	08:05 01 h10 m	−1° 10°	12:50 01 h30 m	12:30 01 h30 m	03 h15 m 01 h35 m	19:00 02 h20 m	18:40 02 h05 m	02 h55 m 01 h20 m
*Both combined*										
Average Variability	2s({*x*_*i*_}) 2s(*x*)	08:05 01 h20 m	07:40 01 h30 m	−4° 11°	12:50 01 h25 m	12:30 01 h25 m	03 h25 m 01 h30 m	19:05 02 h20 m	18:45 02 h00 m	02 h50 m 01 h25 m

For each subset the table lists local time (*t*), mean solar time (*τ*), distance (Δ*t*_*w*_) to winter sunset (lunch and dinner) and winter solar elevation angle *z*_*w*_. Times have been rounded to the nearest five-minute mark except Irish data which have been rounded to the nearest quarter of an hour, angles have been rounded to whole numbers. Data are shown in Fig. 4. Bivariate correlations are reported on Table 9.

Results on bivariate correlations for European eating times are listed in Table 9. Dinner times are strongly synchronized to the winter terminator for a wide range of latitudes with *p* = 38.1(65) min/h (a 4/15 excess of *α*) and variability is reduced by 40% when considering Δ*t*_*w*_ instead of *τ*. Lunch times, on their hand, can be boxed either meridionally (vertically) in a one hour strip centered at 12:30 with lunch meaning eating at noon. Or they can be boxed non-meridionally in a one hour strip centered 03 h30 m before winter sunset with lunch meaning eating with enough ambient light conditions. Data report similar results on the meridional *τ* vs *D*_*w*_ and non-meridional Δ*t*_*w*_ vs *D*_*w*_ testings showing that is hard to elucidate which synchronizer —noon or winter sunset— is stronger.

**Table 9:** Bivariate correlations for lunch and dinner time marks and control variables for European countries.

Europe	Lunch	Dinner
**Meridional quantity**	*y* = *τ*	
vs *x* = *D*_*w*_*r*^2^	0.19	0.657
slope*p*(*u*_*p*_)	$\mathrm{15.7(77)}\,\,{\rm{\min }}/{\rm{h}}$	$\mathrm{38.1(65)}\,\,{\rm{\min }}/{\rm{h}}$
reference*y*_ref_	12:40	19:00
variability2*s*({*y*_*i*_})	01 h25 m	01 h45 m
observations*N*	20	20
**Latitude prone quantity**	*y* = Δ*t*_*w*_ (to sunset)	*y* = Δ*t*_*w*_ (to sunset)
vs *x* = *D*_*w*_*r*^2^	0.16	*0.0809*
slope*p*(*u*_*p*_)	$-\mathrm{14.2(77)}\,\,{\rm{\min }}/{\rm{h}}$	$\mathrm{8.1(65)}\,\,{\rm{\min }}/{\rm{h}}$
reference*y*_ref_	−03 h20 m	+03 h00 m
variability2*s*({*y*_*i*_})	01 h25 m	01 h05 m
observations*N*	20	20

The meridional quantity is mean solar times (*y* = *τ*) at main meals and the latitude prone quantity is distance to winter sunset Δ*t*_*w*_ at main meals. Either case the quantity is tested against shortest photoperiod *D*_*w*_. Each test reports Pearson’s *r*^2^ correlation coefficient (italicized in stationary results), slope and its uncertainty *p*(*u*_*p*_), the predicted value *y*_ref_ at the level *D*_*w*_ = 8 h (*ϕ* ~ 50°) and the variability of the tested variable measured as twice its sample standard population. Last two items were rounded to the nearest five-minute. Uncertainties apply to the least two significant digits. Data are listed in Table 8 and shown in Fig. 4. Polish lunch time and Norwegian dinner time did not enter in the correlation analysis.

Finally relevant times for the location “out of home” (employees only) are listed in Table 10 and bivariate analysis is reported in Table 11. Leaving home time mark occurs at the end of nautical winter twilight and is correlated to labor start time: laborers readily get up, leave home and get to work in the early hours of the morning. Coming home time marks spans along some hours when expressed in local time (see 2*s*({*t*_*i*_}) in Table 10). Contrastingly, they lie within a one-hour strip centered at 2 h10 m after winter sunset with $2s(\{{\rm{\Delta }}{t}_{{w}_{i}}\})=50\,{\rm{\min }}$ and *r*^2^ = 0.136. Therefore the winter terminator seems to synchronize this location.

**Table 10:** Leaving home/coming home time marks obtained from the shares of employees not located at home as a function of time; *t* stands for local time; *τ* is mean solar time, Δ*t*_*w*_ is time distance to winter sunset; and *z*_*w*_ is the winter solar elevation angle at the event.

Country	Label	Leaving Home			Coming Home		
		*t*	*τ*	*z*_*w*_	*t*	*τ*	Δ*t*_*w*_
*Time Use Surveys (employees in weekday only)*							
United States	USA	07:00	06:50	−5°	18:50	18:40	+01 h55 m
Spain	ESP	07:45	06:30	−9°	20:30	19:20	+02 h40 m
Italy	ITA	07:30	07:20	−3°	19:15	19:05	+02 h35 m
Canada	CAN	07:20	07:00	−7°	18:20	18:00	+01 h40 m
France	FRA	07:35	06:45	−10°	18:55	18:05	+01 h55 m
United Kingdom	GBR	07:45	07:35	−5°	18:15	18:05	+02 h15 m
Denmark	DNK	07:30	07:15	−9°	17:30	17:15	+01 h45 m
Average Variability	2s({*x*_*i*_}) 2s(*x*)	07:30 30 min	07:05 45 min	−7° 5°	18:50 01 h55 m	18:20 01 h25 m	02 h10 m 50 min

Times have been rounded to the nearest fifth-minute mark except Irish data which have been rounded to the next quarter of an hour. Simple descriptive statistic values (sample average value and twice sample standard deviation) are listed. Bivariate correlations are reported on Table 11.

**Table 11:** Bivariate correlations for leaving home/coming home time marks and control variables.

Employees	Leaving Home	Coming Home
**Meridional quantity**	*y* = *τ*	
vs *x* = *D*_*w*_*r*^2^	0.415	0.714
slope*p*(*u*_*p*_)	$-\mathrm{16.7(89)}\,{\rm{\min }}/{\rm{h}}$	$\mathrm{40(11)}\,\,{\rm{\min }}/{\rm{h}}$
reference*y*_ref_	07:10	18:05
variability2*s*({*y*_*i*_})	$45\,\,{\rm{\min }}$	01 h25 m
observations*N*	7	7
**Latitude prone quantity**	*y* = *z*_*w*_	*y* = Δ*t*_*w*_ (to sunset)
vs *x* = *D*_*w*_*r*^2^	*0.0496*	*0.136*
slope*p*(*u*_*p*_)	0.7(13) °h^−1^	$\mathrm{10(11)}\,\,{\rm{\min }}/{\rm{h}}$
reference*y*_ref_	−7°	+02 h05 m
variability2*s*({*y*_*i*_})	5°	$50\,\,{\rm{\min }}$
observations*N*	7	7

The meridional quantity is mean solar times (*y* = *τ*) at leaving home/coming home marks, and the latitude prone quantity is winter solar elevation angle *z*_*w*_ or distance to winter sunset Δ*t*_*w*_ at these time marks. Either case the quantity is tested against shortest photoperiod *D*_*w*_. Each test reports Pearson’s *r*^2^ correlation coefficient (italicized in stationary results), slope and its uncertainty *p*(*u*_*p*_), a reference value *y*_ref_ at the level *D*_*w*_ = 8 h (*ϕ* ~ 50° and the variability of the tested variable measured as twice its sample standard population. Last two items were rounded to the nearest five-minute. Uncertainties apply to the least two significant digits. Data are listed in Table 10.

**Figure 5:**
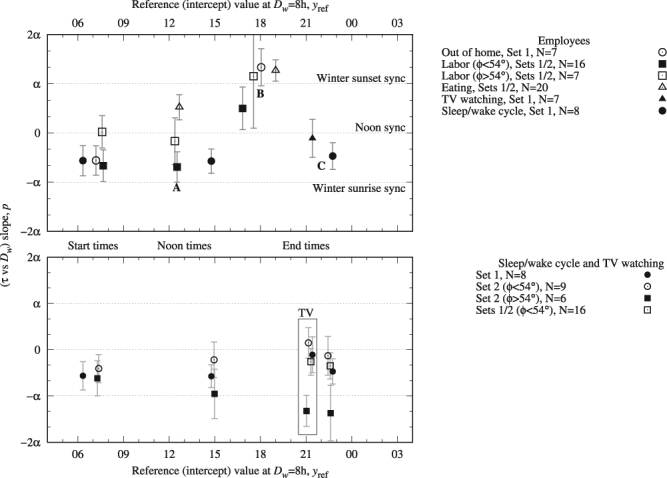
Top panel: slopes *p* for the bivariate analysis *τ* vs *D*_*w*_ as a function of the reference time *y*_ref_ (intercept value at *D*_*w*_ = 8 h) for employees. Vertical axis displays units of *α* = 30 min/h the gradient of winter sunset time vs *D*_*w*_. Zero slope yields meridional behaviour synced by noon, ±*α* slope punctuate winter terminator synchronization. From A (noon times) to B (evening times) synchronization is overturned; then from B to C (night times) synchronization changes again. Bottom panel displays sleep/wake cycle events and TV prime time for a series of statistics. Set 1 refers to national time use surveys reporting data of employees; set 2 refers to Hetus webtool data, reporting standard population data. Reference value for *ϕ* > 54° was located at *D*_*w*_ = 6 h.

Bivariate analysis results for *τ* vs *D*_*w*_ are summarized on Fig. 5 where slopes *p* are presented as a function of the reference intercept value *y*_ref_, which is to say in a chronology at *D*_*w*_ = 8. Vertical axis display units of *α* = 30 min/h, which helps identifying noon and winter terminator synchronizators.

### Average daily consumptions

Average daily consumptions fall in another category of data as they are not related to clock-time, time zones and longitude, but to stopwatch: a duration of time. They represent the shares of one day consumed by an activity.

The shaded area in Fig. 1 is the average daily consumption of every activity: it integrates every daily contribution to the activity. For the sleep-wake cycle notice that sleep time is not the straight difference of wake up times and bedtimes: it also includes intra-day sleeping activity (siesta time) and the different paces at which sleep-wake cycle soars in the morning and drops at night. Table 12 lists the daily average consumption of the primary activities —labor, eating and sleeping, the complementary of the wakeful consumption shown in panel (a) of Fig. 1— and watching TV.

**Table 12:** Daily average times of primary activities laboring *L*, sleeping *S* and eating *M*, and TV watching *T*.

Country	Label	Labor duration		Sleep time	Eat time	TV time
		*L*	Δ*L*	*S*	*M*	*TV*
Time Use Survey (employees in weekday only)						
United States	USA	07 h55 m	−01 h35 m	07 h45 m	01 h00 m	02 h40 m
Spain	ESP	07 h45 m	−01 h35 m	07 h30 m	01 h40 m	01 h30 m
Italy	ITA	07 h40 m	−01 h15 m	07 h40 m	01 h45 m	01 h30 m
Canada	CAN	07 h55 m	−00 h45 m	07 h45 m	01 h05 m	01 h45 m
France	FRA	06 h55 m	−01 h25 m	07 h35 m	01 h55 m	02 h20 m
United Kingdom	GBR	07 h25 m	−00 h15 m	07 h45 m	01 h15 m	01 h55 m
Ireland	IRL	06 h45 m	−00 h45 m	07 h30 m	01 h45 m	01 h45 m
Denmark	DNK	07 h00 m	−00 h00 m	07 h05 m	01 h40 m	01 h45 m
Average Variability	2s	07 h25 m $55\,{\rm{\min }}$	01 h00 m 01 h10 m	07 h35 m $25\,{\rm{\min }}$	01 h30 m $40\,{\rm{\min }}$	01 h55 m $50\,{\rm{\min }}$
Hetus pre-prepared tables (standard population)						
Spain	ESP	07 h50 m	−01 h25 m	08 h35 m	01 h45 m	02 h50 m
Bulgaria	BGR	08 h10 m	−00 h50 m	09 h05 m	02 h00 m	02 h50 m
Italy	ITA	07 h35 m	−01 h20 m	08 h20 m	01 h55 m	02 h00 m
Slovenia	SVN	07 h30 m	−01 h10 m	08 h20 m	01 h30 m	02 h25 m
France	FRA	07 h10 m	−01 h10 m	08 h50 m	02 h15 m	03 h10 m
Belgium	BEL	07 h20 m	−00 h35 m	08 h25 m	01 h50 m	02 h55 m
Germany	DEU	07 h15 m	−00 h40 m	08 h10 m	01 h45 m	02 h30 m
Poland	POL	07 h15 m	−00 h35 m	08 h30 m	01 h35 m	02 h25 m
United Kingdom	GBR	07 h25 m	−00 h15 m	08 h25 m	01 h25 m	02 h55 m
Lithuania	LIT	07 h50 m	+00 h40 m	08 h30 m	01 h30 m	02 h45 m
Latvia	LVA	08 h10 m	+01 h30 m	08 h40 m	01 h30 m	02 h40 m
Sweden	SWE	07 h40 m	+01 h35 m	08 h05 m	01 h35 m	02 h10 m
Estonia	EST	08 h05 m	+02 h00 m	08 h25 m	01 h15 m	02 h25 m
Norway	NOR	07 h10 m	+01 h15 m	08 h05 m	01 h20 m	02 h35 m
Finland	FIN	07 h30 m	+02 h05 m	08 h25 m	01 h20 m	03 h00 m
Average Variability	2s	07 h35 m $45\,{\rm{\min }}$	$05\,{\rm{\min }}$ 02 h35 m	08 h25 m $35\,{\rm{\min }}$	01 h40 m $35\,{\rm{\min }}$	02 h40 m $40\,{\rm{\min }}$

**Figure 6:**
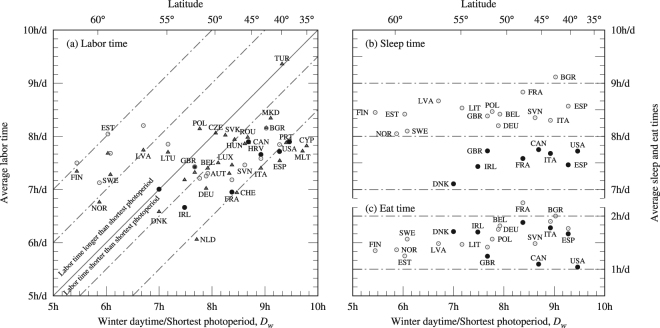
Average daily labor time (left), average daily sleep time (right top) and average daily eat time (right bottom) versus winter daytime. Data from national time use surveys (solid circles), Hetus pre-prepared tables (open circles) and, on left panel, Eurostat (triangles). Two one-hour slanted strips are shown on the left panel: one shows a daily labor consumption 1.5 h longer than the shortest photoperiod; the second one shows a daily labor consumption 1 h shorter than the shortest photoperiod. In the right panels four horizontal strips are shown. Labels (only one for each country in panels (a,c)) indicate ISO-3166-1 alpha-3 country codes. Data are listed in Table 12 and bivariate correlations are reported in Table 13.

Also Δ*L* lists the time difference of the labor time and the shortest photoperiod *D*_*w*_. Hetus data were retrieved from employment daily totals in main activities pre-prepared table taking into account its participation rate, and sleep daily totals in main activities 2-digit level pre-prepared tables. Times have been rounded to the nearest five-minute mark except Irish data which have been rounded to the next quarter of an hour. For each set average value and variability defined as twice sample standard deviation are reported. Data are shown in Fig. 6, bivariate correlations are shown in Table 13.

Bivariate correlations for daily consumptions and *D*_*w*_ are reported on Table 13 for each data subset and both combined; correlations on daily TV time are also reported.

**Table 13:** Bivariate correlations for average daily consumptions versus shortest photoperiod.

	Labor time	Sleep time	Eat time	TV time
*Time Use Surveys* (*employees in weekday only*)				
vs *x* = *D*_*w*_*r*^2^	0.61	0.372	0.086	0.0572
slope*p*(*u*_*p*_)	$\mathrm{24.8(81)}\,{\rm{\min }}/{\rm{h}}$	$\mathrm{8.9(48)}\,{\rm{\min }}/{\rm{h}}$	$-\mathrm{6.5(87)}\,{\rm{\min }}/{\rm{h}}$	$\mathrm{7(11)}\,{\rm{\min }}/{\rm{h}}$
reference*y*_ref_	07 h15 m	07 h30 m	01 h35 m	01 h50 m
variability2*s*({*y*_*i*_})	$55\,{\rm{\min }}$	$25\,{\rm{\min }}$	$40\,{\rm{\min }}$	$50\,{\rm{\min }}$
observations*N*	8	8	8	8
*Hetus pre*-*prepared tables* (*standard population*)				
vs *x* = *D*_*w*_*r*^2^	3.68 × 10^−6^	0.197	0.513	0.298
slope*p*(*u*_*p*_)	$\mathrm{0.0(48)}\,{\rm{\min }}/{\rm{h}}$	$\mathrm{5.8(33)}\,{\rm{\min }}/{\rm{h}}$	$\mathrm{9.5(26)}\,{\rm{\min }}/{\rm{h}}$	$-\mathrm{8.2(35)}\,{\rm{\min }}/{\rm{h}}$
reference*y*_ref_	07 h35 m	08 h30 m	01 h40 m	02 h35 m
variability2*s*({*y*_*i*_})	$45\,{\rm{\min }}$	35 min	$35\,{\rm{\min }}$	$40\,{\rm{\min }}$
observations*N*	15	15	15	15
*Both combined*				
vs *x* = *D*_*w*_*r*^2^	0.0197			
slope*p*(*u*_*p*_)	$\mathrm{2.8(44)}\,{\rm{\min }}/{\rm{h}}$			
reference*y*_ref_	07 h30 m			
variability2*s*({*y*_*i*_})	$50\,{\rm{\min }}$			
observations*N*	23			
*Eurostat Weekly hours*				
vs *x* = *D*_*w*_*r*^2^	0.232			
slope*p*(*u*_*p*_)	$\mathrm{15.2(51)}\,{\rm{\min }}/{\rm{h}}$			
reference*y*_ref_	07 h35 m			
variability2*s*({*y*_*i*_})	01 h15 m			
observations*N*	32			
*All combined*				
vs *x* = *D*_*w*_*r*^2^	0.136			
slope*p*(*u*_*p*_)	$\mathrm{10.0(35)}\,{\rm{\min }}/{\rm{h}}$			
reference*y*_ref_	07 h35 m			
variability2*s*({*y*_*i*_})	01 h05 m			
observation*N*	55			
*All combined and ϕ* < 54°				
vs *x* = *D*_*w*_*r*^2^	0.338			
slope*p*(*u*_*p*_)	$\mathrm{28.8(65)}\,{\rm{\min }}/{\rm{h}}$			
reference*y*_ref_	07 h20 m			
variability2*s*({*y*_*i*_})	01 h05 m			
observations*N*	41			

Each test reports Pearson’s *r*^2^ correlation coefficient, slope and its uncertainty *p*(*u*_*p*_), the predicted value *y*_ref_ at the level *D*_*w*_ = 8 h (*ϕ* ~ 50°) and the variability of the tested variable measured as twice its sample standard population. Last two items were rounded to the nearest five-minute. Uncertainties apply to the least two significant digits. Different sets and combinations are tested for labor time. Table 12 lists values and Fig. 6 shows labor, sleep and eat times.

Figure 6 shows average daily consumptions for primary activities versus *D*_*w*_ (bottom axis) or *ϕ* (top). Vertical axes show fractions of Earth’s rotation period with 8 h d^−1^ ≡ 1/3 and 6 h d^−1^ ≡ 1/4. Average labor time and average sleep time roughly account for one third of a day each.

In panel (b) sleep time from national time use surveys (solid circles) and Hetus pre-prepared tables (open circles) are shown. Although Hetus pre-prepared table reports the combined daily rhythm of “sleeping and other personal care” as discussed previously, it separately lists daily sleep time and other personal care daily time. The latter lies in the range of 40 min, a 7% of the combined daily time.

Panel (b) shows the differences between standard population (Hetus, open symbols) and laborers (Time Use Surveys, solid symbols) which amounts to one whole hour less of daily sleep times. Table 13 shows a weak trend (*p* = 5.8(32) min/h ~ −1 min/°; *r*^2^ = 0.197) with variability 2*s* = 20 min for Hetus data. Variability is 7% of the reference value. Employees in a week day yields similar figures but one hour less of sleep time and smaller variability (5% of *y*_ref_). If Danish datum is excluded employees data show no trend with *D*_*w*_: *r*^2^ = 0.05, *p* = 2.3(45) min/h, 2*s* = 15 min. The lack of a significant latitudinal trend is understandable on the basis that sleep time is an indoor activity and that sleep time is likely a human universal.

In panel (c) daily eating consumption is presented. In this panel French, British, Italian and Spanish data stack vertically, label is displayed once. Eat times lie in the range of one to two hours —a tiny fraction of one day— with remarkable latitudinal trend. For Hetus data *p* = 9.5(25) min/h ~ −1.75 min/°; *r*^2^ = 0.513) with eat times decreasing with decreasing photoperiod. Hetus eat time variability is *δy* = 35 min which is a 35% of the daily eat time. Notwithstanding this, national time use surveys on laborers display opposite trend with meal times increasing with decreasing photoperiod due to the influence of the American data. Table 13 also reports bivariate correlations for daily TV time which show little correlation with latitude.

Panel (a) in Fig. 6 shows average daily labor consumption, which shapes the life of laborers. In this panel a third set of data has been included: the average number of weekly hours of work (converted into daily hours assuming a five day week) in main job obtained from Eurostat database^32^ (triangles) and referred to the year 2016. Again, data sets stack vertically and labels are displayed once.

As well as panels (b) and (c), panel (a) in Fig. 6 can be read horizontally with most of data lying in the range of seven to eight hours a day, irrespective of latitude. Table 13 reports bivariate correlations for labor time and the subsets of data. It also reports all of them combined. Results vividly depend on data set or combination. Hetus data report lack of latitudinal dependence (*r*^2^ < 10^−5^) while national time use surveys soars to *r*^2^ = 0.61 most likely due to small sample size. Eurostat data consists of *N* = 32 countries (Iceland datum has not been considered) and finds correlations (*p* = 15.2(50) min/h; *r*^2^ = 0.232) with 2*s* = 75 min for the full range of latitudes, a 15% of *y*_ref_ = 7 h35 m.

Notwithstanding this, Fig. 6 shows slanted lines at a slope *β* = 60 min/h highlighting a one-to-one coupling between the shortest photoperiod and labor time. They provide an alternative insight: above 54° latitude, daily labor consumption is 1 h to 2 h longer than *D*_*w*_; in contrast, below 54° latitude, most data can be boxed in the range of 30 min to 90 min shorter than *D*_*w*_. Table 13 reports bivariate correlations for *ϕ* < 54° showing an increase in *r*^2^ to 0.338 and slope *p* = 28.8(64) min/h, which is roughly $\frac{1}{2}$ of *β*. It is remarkable considering the myriad of factors unrelated to light conditions that may punctuate labor time: productivity, gross domestic product or shares of economic sectors among others.

## Discussion

Meridional properties characteristically occur at a given instant along a meridian being solar noon the most remarkable of them. Mechanical clocks, conveniently tied to Earth’s rotation, take advantage of that and render time as a distance to noon, or to midnight.

Labor start time marks are not a meridional observation in the range 35° to 54° latitude (see Fig. 2 and Table 3). It is likely that people living along a meridian on this range of latitude and different countries do not get to work simultaneously. Instead, year round, they will likely get to work at the latest sunrise time of the year, Fig. 2 show. To put it shortly, people living at 38° may not feel the necessity of waiting until the Sun has risen at 54° to start working. Correspondingly people at 54° do not feel the necessity of start working once the Sun has already risen at 38°.

Clock hours (7 am/8 am/9 am) synchronize events like labor start but their significance —too early, too late or fine— is punctuated by light conditions —distance to the terminator, not distance to noon— and influenced by latitude. People make this translation and make rationale decisions accordingly populating the best choice. In this process laborers may accept a small winter penalty score in labor start times with the daily outcome of earlier labor end times and the seasonal outcome of start times closer to summer sunrise.

Likewise people living at 54° are likely to agree leaving work earlier, agree coming home earlier or agree having dinning earlier than people living at 38°. All of these processes are likely synchronized by winter sunset time, specially in Europe.

Notice that the rationale for winter synchronization are hardly symmetrically transposed on summer because photoperiod (*D*_*s*_ ~ 17 h at 54°) is exceedingly larger than average labor time. People living at 54° seldom feel the necessity of start working earlier than people leaving at 38° despite they will be observing earlier sunrise. In the same way people living at lower latitudes than 35° are less likely to be driven by the winter sunrise time as it increasingly exceed average labor time. Also the gradient of the terminator with latitude decreases as latitude decreases, which should extinct the role of latitude in the light-dark cycle.

As a general rule searching for latitudinal trends in time marks which span within one hour would require the span of latitudes be large enough the shortest photoperiod spans by two hours. At lunch time this issue is further complicated because competing synchronizers appear. People living along a meridian covering a wide latitude range are likely to have lunch simultaneously around noon (the first synchronizer). And they are also likely to break that behavior and have lunch three hours before winter sunset time (the second synchronizer), Fig. 4 and Table 9 show. Competing synchronizers do not appear at labor start times or dinner times because there is no other significant natural event.

The rationale of the winter synchronizer is: once labor daily rhythm fits the worst case scenario, it only improves as the year progresses and the Sun apparently grows up. That way, and within some variability, labor daily rhythm is linked to ambient light conditions, with societies abhorring labor activity in darkness and harvesting insolation to produce goods. Lack of seasonality is implicit in this idea: if year round time schedules are promoted then winter arises as the worst case scenario and as a synchronizer. Therefore seasonality is another issue which merits discussion.

In ancient times, with time reckoning bound to sunrise and sunset, seasonality in human activity should be expected to some extend. It lost significance as mechanical clock —bound to noon and not showing seasonality by itself— started driving human activity, industrialization lead to decreased labor times and year round time schedules gained popularity. Seasonality was introduced in mechanical clocks (and in time schedules tied to them) by Daylight Saving Times (DST), which is the main, if not unique, source in seasonality nowadays.

DST breaks the seasonal symmetry of the solar activity in a very specific way: sunrise local time variance decreases while sunset local time variance increases; alternatively, time schedules are advanced one hour solar time in summer. Hence DST inhibits “local time” morning seasonality (the appetence of laborers for earlier time schedules in summer) by forcing “mean solar time” morning seasonality. An important point is that societies would have likely abhorred breaking the symmetry the other way around —narrowing the span of sunset times, enlarging the span of sunrise times— because sunrise time is the prime synchronizer; not sunset times and not noon time.

Figure 1 (panel c) shows labor daily rhythm soaring in the morning as laborers start working. The daily rhythm integrates the geographical distribution of laborers and their preferences associated to this factor; their seasonal preferences because it is a yearly averaged quantity and their daily preferences.

One may consider the elapsed time 〈Δ*τ*〉 between the daily rhythm overshooting two convenient thresholds: 25% and 75% of daily maximum activity. That quantity shows an average value 〈Δ*τ*〉 = 80 min (a reasonable time scale for labor activity to daily rise in a society and shorter than seasonality in sunrise local time) with 2*s* = 30 min and uncorrelated with latitude (*r*^2^ < 0.01). All this agrees with low seasonality in modern societies. Understandably similar figures can be obtained for the sleep-wake daily rhythm of laborers.

The calendar date when DST is set on and off also points towards the synchronizing role of winter sunrise. These dates depart from winter solstice date way enough so that the winter sunrise local time still be the latest year round and prevent labor start times from occurring before sunrise. DST was first used in 1916 during the Great War and then in the interwar period. Therefore, by then, time schedules may have been already arranged in a globally similar way to contemporary’s: start times close to winter sunrise time and lack of seasonality.

The survival of DST to the following winter puts time schedules into a major stress because winter sunrise time is delayed by one hour local time—alternatively winter time schedules are advanced by one hour—. The important decisions that were rationally based on daylight (the fundamental zeitgeber), then may become irrational. It was not until 1938, during Spanish Civil War, and soon later in 1940 during World War Two, that DST survived to the following winter in many belligerent and non-belligerent countries. The survival of DST to the following winter is a *time zone advance*. War economy promotes time zone advance, but during peace time they often fail to prevail. Examples of contemporary societies that could not sustain time zone advances for too long are: United Kingdom (1969–1971), Portugal (1967–1976 and 1992–1996), Russia (2011–2014) and Chile (2015). Darkness at labor start time is often the issue.

In contrast, time zone advance in the Canadian province of Saskatchewan (1960), Iceland (1969) and Alaska (1983) succeeded: people traded dark winter labor start times with daylight winter labor end times, a preference which is also observed above 54° in Fig. 2 and discussion thereof. That has also been the motivation of the recent (2017) time zone advance in the Chilean region of Magallanes, the polar most in the country. In every of these four regions absolute latitude is above 50°.

The Chilean case instructs the role of latitude in this issue. Noon occurs almost simultaneously everywhere in Chile and if labor times do not change regionally in Chile they would stack vertically in Fig. 2. However it takes two hours for the winter and summer terminator to sweep the country. As a result both labor start and end times in Magallanes may likely occur in the winter darkness with *z*_*w*_ < −6°. In May 2017 the time zone offset for this region effectively shifted from Δ = −4 h to Δ = −3 h so that labor end times will tend to occur closer to the winter sunset. The rationale for this time zone split is the winter terminator (latitude), instead of noon (longitude).

France, Belgium and Spain, where time zone kept advanced at the end of World War Two (see *δ* > 30 min in Table 1), fall in another category. In the wake of this turmoil the change survived simply because population quickly offset the advancing of time zone by delaying time schedules as historical records can track^33,34^. This trade is more evident in Spain because of its Southwestern most location and can be observed in Tables 2, 4, 6, 8 and 10 by late local times. Mathematically they traded *t* in Equation (5) after Δ changed in a way that *τ* remained. Although this poses no harm for the population it jeopardizes the comparison of time schedules: if local times are to be compared, one whole hour should be subtracted to clock readings in these three countries—or in any other region with advanced time zone—. As an example morningness-eveningness tests can lean toward the eveningness^35,36^ if this rule of thumb is not taken into account. Strictly speaking this is a “clock-time” eveningness, and not a light/dark preference.

The shortest photoperiod sets a significant non-meridional unit of time for life: people at 38° latitude never live a photoperiod shorter than 9 h30 m as people at 55° (*D*_*w*_ ~ 7 h) latitude do. That makes a difference and influences the decision-making process that shapes human activity, notably the labor daily rhythm. Figure 6 (panel (a)) shows working population above 54° must get used to the idea of doing activities in the winter darkness (*z*_*w*_ < −6°) as observed in Fig. 2. On the contrary, below 54° working population can accommodate their duties to winter daytime. Adding a reasonable lunch break, laborers struggle with darkness only in winter labor start time or in winter labor end time, but not both (see Fig. 2 and *z*_*w*_ in Table 2). The far end of the strip highlights an average daily labor consumption 1.5 h shorter than winter daytime. In this scenario individuals and societies can find more ways to accommodate duties to light conditions.

For the purpose of daily rhythm comparisons across time use surveys it should be stressed the existence of a point during the photoperiod that turns the winter sunrise trend characterizing morning activity into the winter sunset trend, which characterizes evening activities (see Fig. 5). This is synchronization overturning. For the labor daily rhythm it happens in the afternoon when light conditions are steady and comfortable. Another overturning should occur at late night when light conditions are again steady.

The first overturning can be described observing the similarity between labor start and labor noon slope. This is showing that at labor noon time mark cultural, social or even political differences have not yet grown enough to break the morning trend set by winter sunrise. Hence, morning rhythms are similar from country to country and therefore it is afternoon rhythms that must proceed in fairly different ways so as to turn the trend upside down. Nature provides a clue: in winter, noon comes later in the morning as latitude decreases; yet, sunset is still further apart. These natural conditions may promote differences in decisions across countries and individuals: morning, afternoon and split shifts as an example. It also accounts for the variability in lunch times, lunch breaks and shares of employees having lunch at home. The excess of shortest photoperiod with respect to labor time should play a significant role. The position of the relative maximum in the labor daily rhythm observed after lunch break (see Fig. 1 panel (c)) shows a meaningful restrictions: it always happen at least one hour before winter sunset. Therefore, in an statistical sense, laborers may find comfortable extending lunch break as long as they do not get back to work in the darkness, the abhorred circumstance.

Another synchronization overturning happens likely at late night. It should be noted that labor noon times occur some two hours before wakeful noon times (see Fig. 5). Hence labor activity leans to “morning” wakeful with “afternoon” wakeful prone to non-working activities. Evening activities are tied to the winter sunset and should sooner or later anticipate the following morning labor activities synchronized to the winter sunrise. The first example is TV prime time marks, mostly synced to noon (see Fig. 5) so that individuals living on the same meridian and different latitudes are likely to be watching TV simultaneously.

An answer to whether sleeping times are sync to noon or not is less definite due to the differences in data for standard population and laborers. The latter set yields time marks following winter sunrise trend (bold circles in Fig. 5). Time marks for the former set seem more synced to noon below 54° (see open circles in bottom panel of Fig. 5). Besides, previous results for Mid Sleep on Free days (MSF) —the half-way point between sleep onset and sleep end, close in meaning to wakeful noon reported here for time use surveys— a chronotype for standard population reported^8^ correlations with summer sunrise time for people in Germany (whose territory cover 10° in latitude, 40 min in time offset and 60 min in shortest photoperiod). Therefore they would also correlate to winter sunset time and it will exhibit positive value of slope in Fig. 5.

Notwithstanding this, the remarkable coincidence of laborer and standard population bedtimes also suggests that they could be distributed irrespective of latitude (see Fig. 3), as it is the case for the most relevant activity preceding bedtime: TV watching. Figure 5 show in light squares this merger of standard population data and employee data for bedtimes and TV prime time. In that case the synchronizer overturning would make people living at lower latitude advance their bedtime— despite they would have come home, have had dinner later— because they should get up earlier. Correspondingly people living at higher latitudes may delay their bedtimes despite they would have arrived home, have had dinner earlier: they will get up later. Both behaviors would result in synchronization overturning. Further investigation will be needed to address thoroughly this hypothesis.

## Conclusion

This work shows that year round time schedules have set the winter day —the day with shortest photoperiod and with roughly the latest sunrise and earliest sunset— as a source of synchronization for labor daily rhythm. It is the worst case scenario year round to which society would find accommodation.

Variability related to cultural or inherited habits is still present, differences between American and European data show. This variability may be linked to latitude in the sense that the longer the shortest photoperiod, the more pathways societies can test.

Since the shortest photoperiod is determined by latitude, this property play a role in understanding social time albeit it is alien for clocks. It is unlikely that a time mark in the morning, 8 am for instance, would play the same role along a meridian because depending of latitude and calendar date it may mean whether the Sun has already risen or not. The same applies in the evening but not at forenoon, noon and afternoon.

This work suggests that the winter sunrise, triggers the decision-making process by which societies within the range 38° to 54° quickly come from the background state of rest in the darkness to the activity during photoperiod. Laborers get up, leave home, get to work quickly in its neighborhood year round, with DST as the only significant source of seasonality. While weak correlations can be found the main evidence is linked to placement: labor start times occur within the winter civil twilight.

Winter sunrise synchronization is overturned in the afternoon as the earliest sunset of the year, the winter sunset, gains significance. Data suggest that the winter sunset, trigger the opposite process in which individuals start making decisions to shut down labor activity. Time use survey data can then track employees leaving work close to winter sunset time, returning to home some two hours after winter sunset, and then having dinner three hours after winter sunset time. Contrastingly, available data shows that TV prime times are irrespective of latitude suggesting a synchronization overturning at late night.

The analysis of sleep-wake cycles does not lead to definite conclusions. Sleeping time of laborers seem to be understandably linked to winter sunrise time while sleep-wake cycle of standard population seems to be irrespective of latitude. It is an open question for future analysis to address this issue.
